# Experiences of pain among Palestinian advanced cancer patients: a socio-cultural reading of reports from the Israeli occupied West Bank

**DOI:** 10.3389/fpsyt.2025.1536839

**Published:** 2025-04-23

**Authors:** Rita Giacaman, Weeam Hammoudeh, Suzan Mitwalli, Abdullatif Husseini, Richard Harding

**Affiliations:** ^1^ Institute of Community and Public Health, Birzeit University, Birzeit, Palestine; ^2^ Florence Nightingale Faculty of Nursing Midwifery and Palliative Care, King’s College London, Cicely Saunders Institute, London, United Kingdom

**Keywords:** cancer, pain, palliative, Palestinian, occupied Palestinian territory, *Tawwakul*

## Abstract

**Introduction:**

This paper explores how pain is conceptualized, defined, expressed and managed among Palestinians with advanced cancer living in the Israeli occupied West Bank of the river Jordan.

**Methods:**

Utilizing qualitative methodology, the study was conducted in three Palestinian governmental hospitals located in the north, center and south of the West Bank. We used a socio-cultural lens which frees our writing from subjugation, and exposes further the need to continue decolonizing knowledge production. Verbatim colloquial Palestinian Arabic quotes obtained from research participants were extracted and translated to English with a focus on meaning rather than semantics, as meaning is deeply embedded in culture. These quotes were then integrated into the text to illustrate the identified themes and subthemes accompanied by selected information about the participant including age, gender, residence, and cancer diagnosis to provide context. We have included in the text the Arabic colloquial terms written in both Arabic and English.

**Results:**

Two dimensions of pain were reported: physical pain due to the effects of the cancer and its treatment, and existential pain, which we defined as the sum total of the human experience of having and dealing with cancer physically, psychologically, socially, economically and spiritually. In addition to treatment with cancer medications, participants emphasized that social support and solidarity from families, friends, neighbors and their community play an important role in helping them come to terms with their illness and pain, and standing by them during difficult times.

**Discussion:**

This social support/social solidarity, is generally regarded as a *wajib* (واجب), or obligation and duty people must fulfill and cannot be neglected. Dependence on God (*Allah*) and *Tawwakul*, that is, the reliance on *Allah*, which they drew upon for support and endurance, was also emphasized. The notion of *Tawwakul* and reliance on *Allah* is of particular importance in assisting patients and their families in coming to terms with their sickness and pain, and in confronting death, as revealed by our participants. However, the incorrect interpretation of *Tawwakul* as fatalism is rooted in colonial and racial perspectives, and needs to be addressed and undone in the process of decolonizing knowledge production.

## Introduction

Pain is a universal human experience that is understood, experienced, communicated, and managed differently in various societies and cultures (1, 2). It is described as a ‘complex multidimensional construct’ with biological, psychological, and social components (3). A clear definition of what constitutes pain is still lacking, as pain is thought of as a subjective experience with measurement relying on verbal concepts (4). Moreover, the expression of pain varies depending on the context in which it is experienced with physical, psychological, social, and spiritual factors contributing to pain expression (5) (6). Thus understanding the cultural experience and expression of pain is essential to its assessment and management. This complexity necessitates socio-cultural ‘competence’ in pain management, that is, person-centered assessment and treatment that are sensitive to socio-cultural beliefs and practices, conceptualized, planned and delivered within the ethno-cultural and socio-economic contexts in which people live with, and manage, their pain (7).

Cancer-related pain is a major cause of suffering. There are variations in incidence and severity observed by race, ethnicity and by sex (8), and it is estimated that around 80% of those with advanced cancer experience moderate to severe pain (9). Although cancer will be the leading cause of serious health-related suffering at the end of life globally by 2060 (10), the availability of analgesics is limited in many parts of the world (11). Evidence points to the presence of inequalities in pain identification and management, for example, by clinicians inadequately recognizing and treating pain among women of color (6, 12). Such inequalities disproportionately affect marginalized communities (13), resulting in an unequal burden of pain among them. Yet medical training arguably continues to prioritize physiological systems and pharmacological interventions (13), and pain is not adequately taught as an experience with physical, psychosocial and spiritual dimensions (14). This points to the need for wider evidence of pain experience and expression among such populations in order to inform health policies and improve access to pain relief (15).

With the study we completed aiming to describe experiences of advanced cancer and its treatment among Palestinians living in the Israeli occupied West Bank of the river Jordan, this paper focuses on exploring how pain is conceptualized, defined, expressed and managed. Questions related to impediments to cancer care access due to significant structural constraints imposed by the ongoing Israeli military rule of the West Bank, including the blockade of roads hampering the movement of people and goods, and creating serious difficulties among patients and all the Palestinian population in general in reaching their destinations, whether for medical care, education, commerce or otherwise, were not addressed in this paper. Likewise those related to the inadequate management of the Palestinian health care services were also excluded since they were also covered in a separate publication (16).

## Methods

This cross-sectional study utilized qualitative methodology with in-depth interviews conducted between September-November 2021. The study was conducted in three Palestinian governmental hospitals located in the north, center and south of the West Bank. We recruited and interviewed 22 patients in total (10 men and 12 women), with an age range of 30-71 years. Nine patients came from the north, three from the center, and ten from the south of the West Bank. Fourteen patients’ cancer was staged at 4, and eight patients at stage 3. The time elapsed between their cancer diagnosis and research interview ranged from one week to 14 years. Five interviews were conducted in an inpatients’ ward, and the rest within daycare.

Inclusion criteria for potential participants (identified by medical oncologists working in the three participating hospitals) were as follows: adults with advanced-stage cancer (stage 3 or 4, as determined by their treating oncologist) of the lung, colon, or breast. These primary cancers were selected had the highest mortality rates in West Bank in 2019 (16). We purposively sampled patients in terms of locality, gender, age and diagnosis. Exclusion criteria were: any other type of cancer or cancer stage 1 or 2 (as determined by their treating oncologist), or patients who were unaware of their cancer diagnosis. Patients were briefed by the study researcher on the purpose and goals of the study, and provided with information sheets in Arabic. Informed consent was obtained orally from participants, including consent to record the interviews (17). This method is appropriate and approved for use in this population, and is in line with the requirements of the Birzeit University Research Ethics Committee Guidelines, given that people locally tend to feel uneasy about signing consent forms in general, preferring the provision of oral consent. The in-depth Interviews lasted between 30 and 90 minutes and were conducted by a researcher from Birzeit University. The full semi-structured topic guide included diagnosis, treatment plan, knowledge about disease and treatment, coping strategies, effects on the patient’s life, the role of the family, pain management, challenges in accessing cancer treatment, communication with the health providers, support accessed, and patients’ recommendations to improve cancer care. All interviews were audio recorded, transcribed verbatim, and files saved on a password-protected computer.

We analyzed the data (transcripts and memos) (18) using thematic analysis (19). We first coded the interview transcriptions and notes, and the codes were then categorized into themes. Afterwards, we arranged themes into a thematic network, which we explored and described, and then summarized, and finally produced interpretative patterns. To strengthen the analysis, we also created analytical memos (20). Quotes were extracted from transcriptions and notes and translated into English. We then structured the results section based on the thematic analysis, analytical memos and our research questions. Each theme is illustrated using verbatim quotes with a participant ID and brief description to demonstrate use of the breadth of sample.

Colloquial Palestinian Arabic quotes obtained from research participants were extracted and translated to English with a focus on meaning rather than semantics, as meaning is deeply rooted in culture. This was conducted by our Palestinian study leads who are bilingual with English as their second language. These quotes were then integrated into the text to illustrate the identified themes and subthemes, accompanied by selected information about the participant including age, gender, residence, and cancer diagnosis to provide context. We have included the Arabic colloquial terms written in both Arabic and English.

Ethical approval was granted by the Research Ethics Committee at the Institute of Community and Public Health, Birzeit University (ref number 2020 3–1) and at Kings College London (ref number HR-20/21-18199).

## Results

While some societies and cultures regard cancer pain as expected and acceptable by patients, professionals, and society as something that patients must endure (21, 22), in our study, pain as a major cause of suffering was reported by almost all participants. In our findings, we organized subthemes along three major themes that emerged: 1) the dimensions of pain experience, and how they are described; 2) pain management, both pharmacological and ‘complementary therapies’; 3) ‘enduring cancer’, with specific focus on pain and the sources of social and spiritual support.

### Dimensions of pain

#### Physical pain

Almost all participants identified their cancer-related pain as a primary concern and the rationale for seeking medical attention. Physical pain was described using powerful metaphors.

For example, a 57 year old man from a village in the Jordan Valley region being treated for colon cancer explained that (17):

“I feel my belly like fire (*ahiss batni zai al-nar احس* بطني زي النار).”).”

This 62 year old man from a northern West Bank city with a rare breast cancer reported that the pain from the cancer was severe (16):

“When you put your hand on it (breast) as if you have hit me with a strong kick, terrible pain, so we want to remove it (the breast).

The wider effect of advanced cancer was on function, with social and financial implications as described by a 40 year old married woman from a village in the north of the West Bank with colon, liver and lung cancer explained (14):

“ I was complaining of things including no energy/vitality/ability to do things (*heil* حيل) … my legs were like dead.”

Most participants also complained about pain related to their medical oncological treatment, including surgery, chemotherapy, and radiotherapy. This 36 year old married man from a central West Bank city with colon cancer reported widespread effects (21):

“… cold air, cold water, cold juice, anything cold affects[the pain] … and becomes like electricity so I have to avoid … I had pimples on all my body, my legs, my hands, my back and my face.”

Other sequelae of treatment included pain and disfiguration which affected psychological wellbeing, as articulated by this 30 year old woman with breast cancer from the north of the West Bank (15):

“ my nails I feel have turned dark, and there is pain in them to begin with … numbness in the mouth and I do not taste food … my psychology)*nafsiti نفسيتي* is affected by) my hair loss…”

A 48 year old married woman from a village in the northern West Bank with colon cancer explained how she keeps having diarrhea when she takes chemotherapy in addition to pain from surgery (19):

“ The first operation was very very difficult (painful) to the point that I went hysterical and I cursed the doctors … I died (*متت* meaning I felt a lot of pain to the point of feeling I was dying) from the chemotherapy, I got tired, tired (meaning sick sick *t’ibt t’ibt تعبت تعبت*)….”

This 38 year old woman from a southern West Bank town revealed (3):

“ the radiation is like burns … and the chemotherapy is (about) nausea (*qalaban mi’deh* meaning the stomach turning *قلبان معدة)* and feeling tired.”

Medical treatment had both physical and psychological consequences. This 59 year old woman from a southern West Bank city with breast cancer explained (2):

“ (side effects) of all types, a human being becomes lethargic (*bisir khoumoul ind a’linsan بصير خمول عند الانسان*), you want to stay asleep and you become nervous/irritable and with a bad psychological state from the chemotherapy of course and want to stay away from all people.’

#### Existential pain and suffering

Given reports that existential pain is ill defined (23), our working definition of this dimension of pain is the sum total of the human experience of having and dealing with cancer; the pain and shock experienced when patients are told they have cancer, which is still understood locally as the kiss of death; the life changing experience of being diagnosed with cancer; confronting the possibility of death and the fear surrounding death or even the desire to die given excessive pain and suffering; going through treatment with a lot of physical and other types of pain associated with it; and worrying about what will happen to family and loved ones. Such existential pain and suffering also includes the respondent’s psychological state and social and economic effects on her/his family and community. In essence, the blend of physical, social, economic, and psychological pain embody the existential pain/suffering that persons people have to contend with when dealing with cancer.

Respondents commonly reported enduring existential pain and suffering, as for example with a 59 year old married woman from a southern city with breast cancer explained (2):

“… I am not afraid of anything, the patient (like her) wishes to die as they would rest from especially the treatment (of the cancer). I expect that all patients like me would wish to die … a person’s body changes, his looks changes … he becomes anxious and angry and cannot take anything … I myself do not like to go to the bathroom to see myself … after I got sick I stayed a month, did not want to bathe … when I remove my clothes, I close my eyes.”

This pain encompasses feelings of sadness over the loss of normal life, in addition to distress for the inability to work or move around as a results of the illness, as this 57 year old man from a village in the Jordan Valley region with colon cancer expressed it (17):

“ By God (*Allah*) (if) I can go back to my normal, to my work, moving around … as it stands now, there is nothing … one is affected when one stays sitting 24 hours (a day) and you are at home doing nothing and you develop complications….I look at myself in the mirror and tell them how I was before and how I am now … I *used* to work on the car (driving the car)… I find myself tied up (*mrabbat* مربط*)* I want to walk and I cannot, meaning (*yaani يعني*) I find myself wanting to fall on my length (*atiih atouli* اطيح عطولي).”

A mix of shock about the cancer diagnosis, confusion and worries about what will happen to one’s children and living in fear as a result was expressed by this 45 year old man from a village in the southern West Bank with colon cancer (6):

“ … it was a very strong shock/hit (*sadmeh qawieh jiddan صدمة قوية جدا*)… and all things were jumbled and interconnected (*ملخبطة ومتداخلة*)…Fear, that is, thinking about the children you have at home … I was raised an orphan and my wife also was raised an orphan, so we lived pain and misery and loss from since we were little.”

Some respondents lamented as they described how this disease and its treatment have resulted in a significant transformations in their lives. This 30 year old married woman with breast cancer from the north of the West Bank but usually lives in the Emirates reported (15):

“ See, I felt that my life has turned upside down (*inqalbat hayati* انقلبت حياتي), I left my work, I left my husband working on his own, like this, meaning (*ya’ni يعني*) I am no longer able to withstand my children, I get tired more, and the children you know … I want someone to help me all the time.”

The psychological toll of the illness, the inability to continue with life like others, the worry about children and family along with an inability to continue in their usual role were reported as interconnected issues. This 48 year old woman from a village in the northern district with colon cancer explained (19):

“ The psychology *(al-nafsieh النفسية*) was most affected that is, what will happen to the children, what will happen to the girls … I do not withstand as before, sensitive, I keep telling them this disease needs more than care … I cry and scream at them … incapable, incapable of working, I touch nothing. I cannot whether on the fire (cooking) or wiping the table. what tires me (what upsets me or what bothers me *illi mit’ibni اللي متعبني*) is this incapacity for work and going on with my life like others.”

This 50 year old woman, married, from a village in the southern West Bank with breast cancer explained this mix of pain and suffering (8):

“ I do not like to be thrown (meaning be sick in bed and not able to do things, *artmi* ***ارتمي***). If I sleep my head explodes, my body gets tired (physically) while I walk, but I feel light and a human being/the daughter of Adam (*bani adameh بني ادمة)*. But (when) I put my head on the pillow. you are like you are dead.”

The cancer can also bring in physical pain with social embarrassment, as this 65 year old married woman from a southern West Bank city with colon cancer revealed (9):

“… as I had that bag (ostomy) I did not go and come back (going out, *arouh wa aji اروح واجي*) at all because it is annoying, embarrassing … sometimes when I am bloated (*manfoukha منفوخة*) and need to pass air. you get bloated like this … how can you at people’s homes?”

This 48 year old man with colon cancer from a southern West Bank town described the social stigma of his diagnosis (10)::

“you discover (*btikshif* or uncover *بتكشف*) people for who they really are … you know who is walking with you for self-interest (*mashi ma’ak la maslaha ماشي معك لمصلحة*.) I was shocked by people.”

Another woman with advanced stage breast cancer from the southern West Bank talked about loss of her sense of femininity/womanhood and estrangement from her husband (2):

“ It became like a separation (from husband). The woman *yaani* feels that she stays away from men, and sexual coldness happens (*bisir burud jinsi* بصير برود جنسي) in the woman’s married life and it leads to a collapse in it (married life). (It is as if) you might encounter someone you do not know and you feel embarrassed in front of them. Finished (*khalas خلص*) because you no longer have the psychological readiness (*ta’ahul nafsi تأهل نفسي*) to be a wife to him as far as your body is concerned (as) there are parts which are missing, the breast.”

### Types of medications used for pain

Nearly all participants indicated that they used one or more analgesic medications to manage pain arising from either the cancer itself or its treatment. Such medications included most notably paracetamol (or acetaminophen in the United States) or ibuprofen (a non-steroidal anti-inflammatory agent) for pain relief, in addition to steroids, calcium to strengthen bones, antibiotics before procedures or if with infection, and various other types of medications to deal with constipation, diarrhea, stomach acidity, hemorrhoids, nausea or diarrhea, vitamins and miscellaneous medications etcetera.

This did not, however, manage all concerns.

A 38 year old woman with colon cancer from a Southern West Bank town complained that (1):

“ yes they gave me laxatives (for constipation) but they caused stomach pain … you would get either diarrhea or constipation, but natural defecation, no.”

Very few participants were prescribed narcotic medications, also called opioids, to alleviate their pain. Only two reported that they were prescribed narcotics. This 59 year old married woman who lives in Hebron City and with breast cancer, reported that she is given paracetamol, a laxative, and hemorrhoidal medications in addition to cortisol, and the narcotic morphine, as well as Femara (letrozole) and Zometa (zoledronic acid) (biologicals) as maintenance medications which induce what she called bone pain (2):

“ one tablet (of morphine) daily … I am allowed up to four. I take four when the pain increases.”

Most participants reported using some sort of herbal/plant products in addition to the modern medical treatment for pain relief. These included boiled peppermint, beets, pomegrenate juice, sugar apple, and linnen seeds, among other herbal and plant products; and were willing to try what their friends and family reported as useful. This 50 year old woman with breast cancer from a town in the southern West Bank revealed that (8):

“ Once they told us about a herb from Nablus (a northern West Bank city). We said all right bring it, and we kept waiting until it came from Nablus, and we found that it is here growing locally (in the southern West Bank town) on the *sanasel* (**عالسناسل,** or hand made stone walls used traditionally among Palestinians).”

The personally initiated, or encouraged by family and friends, use of herb and plant treatment seemed to be common. However, such practices were generally criticized by doctors and nurses, that is, the modern day medical practitioners. This 59 year old woman with breast cancer from a southern West Bank city was sternly told by her doctor that herbal medications are not allowed for patients undergoing chemotherapy (2):

“ at first my relatives would make me drink Indian fungus but did not like the taste. There is a recipe they say, louf (likely *Luffa cylindrica*, with known pharmacological effects against cramps, convulsions, nephritis, asthma and fever among other effects (24)) but I could not accept it … yes, its taste, then the idea that I took from the doctor that whoever is being treated with chemotherapy is not allowed to take herbs.”

The use of curcumin was mentioned by several participants. Evidence suggest some positive effects on patient-reported and treatment outcomes for curcumin in cancer therapy (25) Many participants reported that their doctors told them not to “take risks” with herbs, but this 53 year old woman from a city in the north with metastasized lung cancer explained (13).

“ The doctor told me that he does not believe in such a thing (drinking boiled curcumin) but every week I take a bit of curcumin, I boil it and drink it … that is I get rested (meaning it reduces the pain and makes me rest **برتاح**) when I drink it, it is good.”

Likewise with this 65 year old woman from a village near Ramallah being treated for breast cancer, who was told by people around her that curcumin helps (with the pain) and began to use it but stopped as her doctor told her that (22):

“ there is not certainty in this, all part of experiments, so we cannot take risks (and take the curcumin).”

### Diet

Several participants reported that they were told not to eat sugar by their clinicians, or were advised by others to avoid sugar as a way of dealing with cancer and its pain. A 45 year old man from a southern West Bank village with colon cancer was at first told by the medical staff treating him to avoid sugar, red meat and rice (6);

“At first (I followed) a special diet and avoided sweets, I avoided red meat, rice. ate light meals in the beginning … later, the disease came back and they told me (medical staff) do not eat meat. do not eat (this or that) and afterwards I blew it (*faratitha فرطتها* meaning stopped eating this diet, with the word *farat* meaning unravel).”

A similar situation was noted by a 36 year old man from Ramallah city with colon cancer who was told by the dietician not to eat sugary foods (21):

“ sugar, he told me to avoid chocolates as they strengthen cancer cells, we do not want to strengthen them, we want to weaken them.”

However, the oncologist corrected this information and indicated that patients could eat what they want.

### Enduring cancer: family and communal/social support and spirituality

As described above, many late-stage cancer patients struggle with pain and the broader consequences on their lives. While medications and herbs partially alleviate this burden, most patients highlighted the important roles that social support (from their families and communities), and their faith play in helping them accept and endure their disease and the associated pain.

#### Family and communal/social support

All participants, except one, reported that they receive strong social support from families, friends, neighbors and the community in general. Families, and friends were reported as having big roles in helping the participants go through the pain of knowing they have cancer, adjusting and being helped with pain and disability, financially helping out, and seeking medical care and taking participants to the hospital for treatment and a variety of other chores.

Wives and husbands were reported as the primary sources of support and care. This 48 year old man with colon cancer emphasized how his wife was the main support (10):

“ *Imm Mustafa* (the mother of Mustafa as his wife is called, and as women are called locally after they have their first male child) is the first and last support. *Imm Mustafa mashallah* (*ماشالله* what *Allah* has willed, an expression of something good, or beautiful, and a blessing from *Allah*) is not failing short (*ما بتقصرش ma bit qassirsh*). What helps you too is when you feel that people are standing by you, and ask about you and visit you, it lifts your spirits (*btirfa’ manawiiatak بترفع معنوياك*).”

Moral support (*da’m ma’nawi دعم معنوي*) provided by family members was also reported, as this 36 year old man from a central West Bank villages indicated (21):

“ There is moral support (*دعم معنوي*) in that (his brothers) always ask about me, always want to be reassured about me (*byittamanu alai بطمنو علي*), and tell me not to worry, not to care, a period and it will go and a period and it will pass, *yaani* they give one hope*.”*

This 54 year old man from a southern West Bank village with colon cancer also expressed the family-wide role of support and managing information on behalf of the patient (7):

“ They (his nuclear and extended family) have a big role. They did not let me need to think or anything … they are ahead of me in work, my son, my brother, my family and naturally my in laws. they were proactive and ahead (*sabbaqeen سباقين*) talking to the doctor about the case while I had no idea.”

While families and friends were reported as providing needed social support, children, especially young children were noted as providing a real positive push to the participant, as this 33 year old man from a village in the southern West Bank indicated (5):

“I tell you, the children, I have little ones, those who are giving me an abnormal (positive) push.”

However, this 59 year old woman from a southern West Bank town with breast cancer reported an estrangement from her husband because of the change in her body and breast (2):

“ It became like a separation (from husband). The woman *yaani* feels that she stays away from men, and sexual coldness happens (*bisir burud jinsi* بصير برود جنسي) in the woman’s married life and it leads to a collapse in it (married life). (It is as if) you might encounter someone you do not know and you feel embarrased in front of them. Finished (*khalas خلص*) because you no longer have the psychological readiness (*ta’ahul nafsi تأهل نفسي*) to be a wife to him as far as your body is concerned (as) there are parts which are missing, the breast.”

#### Spirituality and *Tawakkul* (reliance on Allah)

In this study, most participants invoked ideas related to *Allah* and the reliance on *Allah* (*tawwakul توكل*) as the ultimate method of dealing with their pain, and the difficult period in their lives they are passing through. The notion of reliance on *Allah* seemed to be a strong factor in helping participants in withstanding, and also accepting their predicament and managing the different types of pains they are experiencing, as this 47 year old woman from a southern city of the West Bank with breast cancer expressed it (4):

“ What comes from *Allah* is good, whatever is from *Allah alhamdullilah* (الحمد لله - meaning acceptance). We have to be patient (‘*aleina alsabr* علينا الصبر) and help is from *Allah (‘ala Allah al’awn وعلى الله العون*)…This is from *Allah*, and what comes from *Allah* I do not get upset (about) we are slaves and the command is for *Allah*.”

Some reported being upset by their condition at first, but then accepted their plight as the will of *Allah*, as this 65 year old woman with breast cancer from a central West Bank village explaine (22):

“ At first I was upset and until now of course. but I say this is something from *Allah*, *khalas*, this is written (*maktoub* meaning written by divine will, predetermined), and you have to live with the issue, you have to live with the treatment.”

This 36 year old woman from a northern West Bank village with colon cancer, seems to have been appeased by her *Tawakkul (* 18):

“ When they told me it is advanced, I got psychologically tired (sick, *t’ibat nafsiti تعبت نفسيتي*), but *al-hamdullilah* one goes back and entrusts his affair with *Allah* (*yiwakkel amro lillah*).

The occurrence of disease and the accompanying pain is understood as due to the will of *Allah*, and helps in the acceptance of the idea of death as well. A 57 year old man from a Jordan Valley village, with colon cancer summed it up (17):

“ This disease is from the God of the Worlds (*Rab al-’Alamin* (*رب العالمين* and I do not object to *Allah’s* decree. I do not ask *Allah* if he wants to take you to the day of judgment(in) a year, two years … we cannot reproach our *Allah* (*بنقدرش نعتب على ربنا*)

That is, the trust in *Allah* seems to help patients in accepting their fate as a matter of destiny which cannot be changed, as this 57 year old woman from a village in the central West Bank (20):

“ I relied on *Rab al-’Alamin*. how can I change anything. you have to rely (on *Allah*).”

## Discussion

This study aimed to describe how pain is conceptualized, defined, expressed and managed among Palestinians in the West Bank with advanced cancer. We identified notions of pain that participants reported which maintain the coherence of the inseparability of physical and psychological/mental pain. This inseparability is very much part of how health, and pain, are generally understood in the Palestinian context (26). In fact, a range of words are locally recognized and used to refer to health status on a continuum between Ease and Dis-ease which do not separate physical from psychological/mental health. Such words/terms integrate physical and mental health and are used by people to describe their health, such as, for example (in the feminine) wilted (*dablaneh دبلانه)*, low energy or sluggish (*habtah هابطه*), not able or unable to perform (*mish qadreh مش قادرة*), broken/achy (*mkaswara مكسوره*) or even unhappy (*mish mabsuta مش مبسوطة*) when *mish mabsuta* means that a person is not feeling well physically and otherwise, so not merely unhappy as translated to English (27).

We identified two dimensions of pain which were reported by participants: physical pain, and existential pain. Physical pain due to the effects of the cancer (28–30) and pain as a result of cancer treatment (31–33) are commonly reported in the Anglo-saxon literature. However, it is important to emphasize that cancer treatments with cytotoxic agents are not only painful, but they can also have adverse effects which reduce the quality of life of patients, and can be debilitating. They are associated with not only morbidity, but also mortality (kill the cancer cells and kill the patient in the process so to speak). In fact, systemic chemotherapy is known to not only treat cancer by killing tumor cells and/or controlling their spread), but is also associated with severe side effects, including the death of other non-cancerous types of rapidly dividing cells (34–36). This can leave cancer patients with severe side effects and significant pain, as noted by our respondents.

The second dimension of pain, existential pain, has received insufficient attention among researchers (37, 38), and continues to be reported as an ill-defined concept (23). It is worth mentioning that existential pain includes the concerns women have about their bodies and body image, as they witness how their body changes, affecting their mental and physical states and wellbeing. Such changes also affect marital life including having to cope with missing parts, combining physical with psychological and social suffering women endure. However, this existential/suffering pain seems to be problematically understood as largely related to spiritual and distress issues combined, which, as a result, are conflated in their meanings (39). This conflation not only creates confusion, but also obscures how the pain and suffering endured by cancer patients prompts them to resort to spirituality to manage this suffering, which we believe is a separate and significant entity from the notion of existential suffering and pain, and refers to how cancer patients manage this pain and suffering instead. Consequently, we opted for a definition of existential suffering pain as noted above in results, as the blend of physical, social, economic, and psychological pain which embody the existential pain/suffering that persons have to contend with when dealing with cancer, and left the issue of spirituality out of this definition to be included as one of the methods of pain management which patients employ for relief in its own right.

This brings us to the findings related to how such reported pain is alleviated. First and foremost, patients deal with their pain by seeking modern biomedical help by doctors and nurses at hospitals who provide them with modern medications. Whether pain due to cancer or as a result of treatment, participants reported having been prescribed largely the pain medication paracetamol (alternatively called acetamenophen), with limited efficacy in dealing with moderate or severe cancer pain (40); and non-steroidal anti-inflammatory agents such as ibuprofen, which is recommended for use for mild or moderate pain (41). Only two participants reported that they were given narcotic/opioid medications to alleviate the pain, such as codeine or morphine. Yet, patients with late stage cancer often require strong analgesia (42). This insufficient use of opioids is likely to be related to reluctance in prescribing strong opioids (43) and their low availability as a result of what is believed to be widespread over-regulation given fears of dependence (addiction) on the medication not only in the Israeli occupied Palestinian territory, but also in the Arab region in general (44).

Unfortunately, palliative care in the Israeli occupied West Bank is still in its infancy. While some non-governmental organizations provide palliative care services, such services are limited, and a national level program is still absent (45). This was highlighted in the region during the COVID pandemic (46). Moreover, a lack of pain management clinics and specialists coupled with the inadequate recognition of pain management as a priority issue in the national health agenda (47) compound the problem. This situation is exacerbated by the frequent unavailability or supply interruptions of painkillers and other medications required by cancer patients attending these oncology hospitals due to serious structural constraints imposed by continued Israeli military rule of the West Bank and those related to the inadequate management of the Palestinian health care services (16). The need to develop pain management and palliative care services in the West Bank Palestinian governmental oncology hospitals, and among all Palestinian health care facilities, is urgently needed and a priority for action.

Cancer patients and their families are pro-active nonetheless. They do not wait for medications to become available, and in any case try different methods of pain relief other than modern medications prescribed by oncologists, whether such medications are available or not. Herbal medications and medications of local plant origins were also used to alleviate pain. For example, respondents reported using boiled peppermint to relieve their pain. This is a local plant which is commonly used by the Palestinian population and others in the region for stomach ailments. It is known scientifically as *Mentha piperita*, and has antiseptic and carminative properties, which relieve distention/gases and abdominal pain (48). Likewise, several participants reported using the local plant curcumin (كركم) to treat their pain. Interestingly, the biomedical Anglosaxon literature does indicate curcumin’s possible effectiveness in cancer therapy (49); with modern scientific reports revealing that curcumin has anti-inflammatory and antioxidant properties (50). In fact, curcumin has been used in the treatment of a variety of diseases including cancer, pulmonary disorders, anxiety and depression, osteoarthritis, diabetes mellitus and in the treatment and prevention of brain disease, among other uses (25). Yet, despite this modern biomedical evidence, it appears that local oncologists are either hesitant about recommending to patients to use curcumin, perhaps because of possible interactions with cancer medications which have not been tested and scrutinized; or because they are unaware of the biomedical literature indicating curcumin’s effectiveness, or both.

These results point to a triple problem related to the use of herbal medications in the management of cancer. The first is that of the worldwide deficient investigations of effective, and possibly effective, medications of plant origins used in ethnomedicine and their interactions with other modern medication. In the case of late stage cancer the effectiveness of herbal medications against the pain of late stage cancer begs further research. However, it is intriguing to note that medicines of plant origins have long been known and used to treat various sorts of ailments, including, for example, *belladonna* leaves which have anesthetic properties and are used externally to relieve pain and local inflammations; or *Hyoscuamis folia*, or *Jusquiame* leaves, which are also known to relieve pain and are used as sedatives; or even *Digitalis folium*, used to extract the glucoside digitoxin which has been used historically and even currently in some countries to treat congestive heart failure (48). Such disregard for ethnomedicine is attributed to financial, scientific, and other impediments, as well as the limited interest of the pharmaceutical industry in conducting studies of efficacy and potential drug interactions (51). These results call for the decolonization of research production, whereby sufficient funding, training and research can include the various aspects of ethnomedicine or what is called Complementary and Integrative Medicine (52).

The second problem pertains to the limited consideration of modern medical curricula in low and middle income countries, including local medical schools, to ethnomedical substances used for the treatment of diseases. It is maintained that a hierarchy of systems of medicine exists but is not acknowledged. Yet this hierarchy is prevalent in the world, where biomedicine is afforded a top place, with healing traditions of local origins not recognized or minimally so, and with the education of health professionals focused on biomedicalized curricula, thus undercutting local health traditions (53). This is another issue requiring the decolonization of knowledge production, whereby ethnomedicine and ethnomedical substances shown to be effective would take their place and become integrated in curricula with modern medicine. It must also be noted that these problems are also compounded by a third problem, namely the absence of a formal continuing medical education system and limited access to medical journals in the occupied Palestinian territory (54). Such constraints can understandably prompt oncologists to hesitate or even prohibit the use of herbs and plants by patients to relieve their pain and suffering, and to rely on the curricula they were trained in, which could have been 10, 20, or even 30 years ago. Information from medical professionals on dietary requirements is also indicative of inconsistent knowledge and insufficient access to resources, with some medical staff informing patients to avoid sugar, red meat and rice completely, while others reported by this study’s participants as providing the more balanced view of eating such foods in moderation.

All participants except one emphasized that social support and solidarity from families, friends, neighbors and their community plays an important role in helping them come to terms with their illness and pain, and standing by them during difficult times. Such support also includes helping in chores at home, providing financial assistance and helping them in reaching medical services for treatment, among other types of help. This social support/social solidarity, is generally regarded as a *wajib واجب))*, or obligation and duty people must fulfill and cannot be neglected. It is part of the Palestinian *Sumud*, or sticking to the land no matter how difficult conditions are. This *Sumud* is induced by long term chronic warlike conditions and the enduring impact of over a century of Zionist settler colonialism which continues to pose significant threat to the viability of the Palestinian people (55). That is, *Sumud* is part of the Palestinian capacity to endure and resist (56) chronic violation and long term exposure to the Israeli occupier’s colonial violence and threat to survival.

Finally, the dependence on Allah and *Tawwakul*, which participants drew upon for support and endurance was emphasized by all except one participant. The notion of *Tawwaku*l and reliance on *Allah* (57) is part of a spiritual belief system followed by more than one billion Muslims around the world (58) and is of particular importance in assisting patients and their families by coming to terms with their sickness and pain (59), and in confronting death as well, just as has been also revealed by our participants. Muslims believe that death and illness cannot be avoided as they are *Allah*’s will, or written (*maktoub*, مكتوب). Such a belief is known to help people cope with their illnesses and in dying in peace (60). However, *Tawakkul* has two components. The first is the principle of *I’qal* (*اعقل*) meaning do all what is within your means, then *Tawwakal*, meaning once you have done what is in your means then rely on *Allah*.

Unfortunately, *tawakkul* has been problematically misinterpreted as fatalism and passivity for having accepted *Allah’s* will, and of course, inaction in dealing with disease. Such an interpretation of *Tawakkul* as fatalism runs contrary to the findings of this research, and our informed by Palestinian society’s understanding of the concept of *Tawwakul*. Our participants were IN hospital receiving treatment when we interviewed them, and did not exhibit any fatalism as they sought medical help; but at the same time they were relying on *Allah* for support. A recent systematic review of the evidence for the experiences and preferences of Muslim patients and/or families for end-of-life care in Muslim-majority countries found “closeness to *Allah*” was a key theme as well (61).

To be sure, the interpretation of *Tawwakul* as fatalism is rooted in colonial and racial perspectives, and needs to be addressed and undone in the process of decolonizing knowledge production. In his ground breaking work Orientalism (62), the Palestinian public intellectual Edward Said highlighted how orientalists perceive people of the East and Arabs, in mostly negative and dehumanizing ways. They are often portrayed as subjects to be studied and controlled rather than active agents, as passive fatalistic Muslims. Said argued that such orientalist stereotypes and ‘othering’ help in legitimizing colonial control and domination, and serves as a basis for the denial of the Palestinian people’s right to resist Israeli colonial occupation of the West Bank and Gaza Strip.

## Conclusion

The French philosopher Michel Foucault noted that conducting a critique requires the inclusion of what he termed subjugated and disqualified knowledges (63). He described subjugated knowledge as knowledge which exists but has been systematically buried, disguised or omitted, and is not recognized as knowledge in its own right. This is reminiscent of how, in order to publish in Anglo-Saxon scientific journals, we – along with other researchers from other parts of the world – have had the experience of publishing in scientific and other types of outlets, but often only after having accepted and used colonial knowledge, understandings, approaches, and methodologies.

Foucault also explained that disqualified knowledge means inadequate, naïve, low in the hierarchy of scientific legitimacy. This includes popular knowledge (*le savoir des gens*). He emphasized that criticism can only perform its work with the re-emergence of such local popular, yet deemed as low ranking knowledges. This too resonates with the Palestinian experience where our knowledge of our history and our narrative of dispossession, dispersion and chronic and ongoing exposure to colonial violence has long been excluded from scientific discourse and the Western media, and this continues.

Finally, we conducted this study using a socio-cultural lens which frees our writing from subjugation, and exposes further the need to continue decolonizing knowledge production. Whether by insisting on not separating physical from mental health; by describing in as much as we can what patients use from an array of local medical herbs and plants generally not recognized by biomedical professionals (popular knowledge as Foucault would have it); by emphasizing social support and solidarity so crucial for the Palestinian people’s survival; and by rejecting the racist and colonial conflation of the Muslim notion of *tawwakul* with fatalism and providing an alternative explanation which is compatible with the local understanding, we hope to have contributed to the decolonization of knowledge production and assisted in recognizing Palestinian (and other) popular knowledge as knowledge in its own right.

## Strengths and limitations

This study provides a deeper understanding of the personal experiences of late-stage cancer patients, offering insights into the complex dimensions of physical and existential pain and how patients manage these pains. However, the findings may not be generalizable and may not fully capture the diversity of experiences among late-stage cancer patients. Additionally, selection or interview bias may have influenced the results, potentially leading to the overrepresentation of certain perspectives or interpretations of the data.

## Data Availability

The raw data supporting the conclusions of this article can be made available upon request.
